# Study on the radiotherapy effect and serum neutral granulocyte lymphocyte ratio and inflammatory factor expression of nasopharyngeal carcinoma

**DOI:** 10.1515/med-2023-0842

**Published:** 2024-01-16

**Authors:** LiPing Wu, JianPing He, YuQing Zheng, Yang Li

**Affiliations:** Department of Otolaryngology, Huzhou Central Hospital, Huzhou, Zhejiang, 313000, China; Department of Otolaryngology, The 910th Hospital of the Chinese People’s Liberation Army Joint Logistic Support Force, Quanzhou, Fujian, 362000, China; School of Engineering, Huzhou University, Huzhou, Zhejiang, 313000, China; Department of Otorhinolaryngology Head and Neck Surgery, Ningbo Yinzhou No. 2 Hospital, No. 998, Qianhe Road, Yinzhou District, Ningbo, Zhejiang, 315100, China

**Keywords:** clinical target volume, inflammatory factor, intensity-modulated radiotherapy, nasopharyngeal carcinoma, neutrophil lymphocyte ratio

## Abstract

**Purpose:**

To compare target area delineation schemes in intensity-modulated radiotherapy (IMRT) effect on patients with locally advanced nasopharyngeal carcinoma (NPC).

**Methods:**

A total of 88 NPC patients received IMRT and were assigned into control group (*n* = 44) and observation group (*n* = 44) based on MRI and CT imaging. In the control group, the treatment range was determined as the clinical target volume (CTV) as the gross tumor volume (GTV) + 5 mm. In the observation group, high-risk target areas CTVp1 was GTVp + 5 mm, lymphatic drainage area CTVn1 was GTVn + 5 mm, medium-risk CTVp2 was CTVp1 + 5 mm margin + the whole nasopharyngeal area, CTVn2 was CTVn1 + 5 mm. Radiotherapy treatment course was 6–8 weeks, four times a week.

**Results:**

The observation group had higher total effective rate, with fewer complications. Neutrophil lymphocyte ratio (NLR), interleukin (IL)-6, and tumor necrosis factor (TNF)-α levels were lower after radiotherapy in both groups compared to before radiotherapy, with the observation group demonstrating lower levels than the control group. The effective group exhibited lower serum NLR, IL-6, and TNF-α compared to the non-effective group. T stage, target location, serum NLR, IL-6, and TNF-α were risk factors for the effect of radiotherapy.

**Conclusions:**

Serum NLR, IL-6, and TNF-α have predictive significance for radiotherapy effect.

## 1 Introduction

Many tumors, especially head and neck tumors in the early stages, can be cured with radiotherapy. Nasopharyngeal carcinoma (NPC) is an Epstein-Barr virus-associated malignant tumor of nasopharynx. Radiotherapy mode, delineation of clinical target volume (CTV), radiation dose, and course of treatment are important factors affecting the radiotherapy effect and survival outcome of NPC [1]. Intensity-modulated radiotherapy (IMRT) has been shown to be safe and effective in reducing the radiation dose of gross tumor volume (GTV) while minimizing exposure damage to organs at risk, reducing radiation-related complications, and improving quality of life after radiotherapy [2]. Radiotherapy is planned based on MRI and CT imaging [3]. Based on the International Guideline for the Delineation of the Clinical Target Volumes (CTV) for NPC [4], high-risk primary tumor CTV and lymphatic drainage area (radical dose) and middle-risk tumor CTV and lymphatic drainage area (elective irradiation of regional limatics) were determined, and adjacent tissues were delineated according to T stages.

Radiotherapy sensitivity and response are key factors in clinical efficacy [5]. In addition to tumor expression specific molecules [6], we also observed abnormal release patterns of serum neutrophil lymphocyte ratio (NLR), interleukin (IL)-6, and tumor necrosis factor (TNF)-α during radiotherapy, which may have certain indicative effects on radiotherapy effect. Takenaka et al. [7] included a total of 5,397 patients with NPC in nine studies to perform meta-analysis, and found that that NLR exceeding the critical value was significantly correlated with survival outcomes, including overall survival (OS), disease-free survival (DFS), progress-free survival (PFS), and distant metastasis-free survival (DMFS), suggesting that elevated NLR predicted worsening of OS, DFS, PFS, and DMFS in NPC patients. Al-Kholy et al. [8] indicated that serum inflammatory cytokines (IL-6 and TNF-α decreased and IL-1β increased) can be used as predictors of survival in NPC patients after chemoradiotherapy. The increase of serum IL-6 level before treatment is a significant specific predictor of high mortality. The increase of serum IL-1β level after treatment indicates that the treatment effect is good and the survival rate is likely to be improved.

The present study mainly compared the effect of different target area delineation schemes in patients with locally advanced NPC undergoing IMRT, and evaluated the value of serum NLR and inflammatory factors in predicting radiotherapy effect.

## 2 Methods

### 2.1 Object information

We selected 88 patients with NPC (stage IIb–IV) from October 2018 to October 2019. Patient pre-treatment evaluations encompassed a comprehensive assessment of medical history, thorough kidney and liver function tests, and nasopharyngeal endoscopy. The staging evaluation entailed the utilization of MRI and CT scans of the nasopharynx and neck. Additionally, chest X-rays, abdominal ultrasound, and bone scans were conducted to identify any distant metastases. The clinical staging was determined according to the staging system of the American Joint Commission on Cancer. Inclusion criteria: (1) age 18–75, Karnofsky performance score greater than 75, (2) patients exhibiting low tolerance or experiencing severe complications throughout the treatment procedure were deemed unsuitable for further radiotherapy, (3) patients received IMRT treatment and followed up, and (4) patients provided informed consent and ethical medical approval. Exclusion criteria: (1) patients with nasopharyngeal metastasis or other primary tumors, (2) patients with expected poor radiotherapy effect accompanied by serious complications, (3) patients gave up treatment halfway, and (4) patients with severe cardiopulmonary liver and kidney dysfunction who could not complete prescribed radiotherapy courses.

Patients’ names and related information were entered into Excel spreadsheet by random number table method, and random integers were generated by Excel’s Rand function. Subsequently, patients were categorized into two groups in a randomized approach, i.e., control group and observation group, with 44 cases in each group. There was no significant difference in the general clinical data between the two groups (*P* > 0.05). Patient’s information is listed in Table 1.

**Table 1:** Comparison of general clinical data between two groups

Group	Number of cases	Male/female	Age (years)	Body mass index (kg/m^2^)	TNM stage			Maximum tumor diameter (mm)	Hypertension	Diabetes mellitus
					IIb	III	IV			
Control group	44	30/14	52.54ol	25.64ol	16	19	9	3.564ol	10 (22.7)	4 (9.1)
Observation group	44	26/18	54.38vat	25.48vat	14	20	10	3.748va	8 (18.2)	5 (11.4)
*t*/.^2^		0.786	0.569	0.326	0.212			0.264	0.279	0.000
*P*		0.375	0.487	0.647	0.900			0.767	0.597	1.000


**Ethics statement:** The present study was approved by the Ethics Committee of Huzhou Central Hospital and written informed consent was provided by all patients prior to the study start. All procedures were performed in accordance with the ethical standards of the Institutional Review Board and The Declaration of Helsinki, and its later amendments or comparable ethical standards.

### 2.2 Radiotherapy program

Two groups of patients received different target area delineation schemes based on MRI and CT imaging. The scan was performed by Toshiba 72 cm spiral CT machine with 16-row aperture. The scanning range of CT cross-section was 5 cm from head to clavicle, with layer thickness of 3 mm and layer distance of 3 mm. MRI scans were performed using the GESIGNA 2.5T scanner, using a head and neck coil to scan from the middle of the brain to the lower edge of the clavicle. Cross-sectional MRI was performed with T1WI and T2WI, and sagittal MRI was with T1WI, both cross-sectional and coronal MRI examinations were done with fat-saturated fat-suppressed enhanced T1WI. Scanning parameters were set as layer thickness 5 mm, spacing 1 mm, matrix 320 × 224, excitation 2–4 times, T1WI (FSE, TR = 340 ms, TE = 11.5 ms), T2WI (FRFSE, TR = 4,500 ms, TE = 85 ms), and fat-saturated fat-suppressed enhanced T1WI (FSPGR, FLIP = 85 PG TR = 200 ms, TE = 2.5 ms). Image fusion method: CT images and MRI cross-sectional images were transmitted to the OTP workstation for image fusion through the network. First, the brainstem, left and right optic nerve, basilar artery, and dental process were selected as internal references by Landmark method, and then the contors of the head and face reconstructed by Manuel method were used as references for further fine-tuning matching. The fusion effect is determined by the joint evaluation of the team members.

Target area delineation method: According to ICRU50 and 62 reports, the control group received irradiation in CTV (GTV + 5 mm) at radical dose (69.76 Gy/32 times, 2.18 Gy/time). GTV included lymph node metastasis area, and organs at risk included temporal lobe, brain stem, optic nerve, optic chiasma, eyeball, crystal, parotid gland, cochlea, oral cavity, mandible, temporomandibular joint, supraglottic larynx, glottic larynx, cervical segment esophagus, and spinal cord. Organs at risk were irradiated using Elekta Synergy accelerator at 6 MeV X-ray in seven-field according to the dose recommended by Radiation Therapy Oncology Group (RTOG) 0225 report. The observation group was irradiated according to the International Guideline for Delineation of Clinical Target Volumes for Nasopharyngeal Carcinoma in 2017. High-risk CTVp1 = GTVp + 5 mm, and lymphatic drainage CTVn1 = GTVn + 5 mm were irradiated at a radical dose (69.76 Gy/32 times, 2.18 Gy/time). Medium-risk CTVp2 = CTVp1 + 5 mm + whole nasopharynx and CTVn2 = CTVn1 + 5 mm were irradiated according to elective irradiation of regional limatics (54.4 Gy/32 times, 1.7 Gy/time). Organs at risk (PTV1 = CTV1 + 5 mm, PTV2 = PTV1 + 5 mm) were irradiated at 58 Gy/32 times and 54 Gy/32 times, respectively. The range of organs at risk and the dose of irradiation were the same as those of the control group. The radiotherapy cycle of the two groups generally lasted for 6–8 weeks, at least four times a week. During radiotherapy, adverse reactions of patients should be observed closely, and patients with severe radiotherapy complications should be suspended and given symptomatic treatment.

### 2.3 Observation indicators

The total effective rate and radiotherapy-related complications were compared after radiotherapy, and serum NLR, IL-6, and TNF-α levels were compared before and after radiotherapy. The clinical efficacy according to WHO criteria could be grouped as significant, effective, stability, and ineffective [9,10]. Total effective rate = (significant + effective)/total number of cases × 100%. Complications mainly include bone marrow suppression and serious injury of adjacent organs, which are recorded according to RTOG/EORTC (Radiation Therapy Oncology Group/European Organisation for Research and Treatment of Cancer) radiotherapy complications and grading [11,12]. NLR, IL-6, and TNF-α were assayed by ELISA kits (Sigma, USA) and average values were taken after three repeated measures.

### 2.4 Statistical analysis

Data were analyzed by SPSS20.0 statistical software. Measurement data (mean ± standard deviation) were compared by independent sample *t*-test to analyze the difference between the two groups. Paired *t*-test was used to compare before and after treatment. Enumeration data (number of cases or percentages) were compared by *χ* ^2^ test. Receiver operating curve (ROC) was utilized to analyze the accuracy of serum NLR, IL-6, and TNF-α to predict the effect of radiotherapy. Multivariate logistic regression analysis was used to screen risk factors that affect the effect of radiotherapy. The stepwise regression method included 0.10 criterion and excluded 0.05 criterion. *P* < 0.05 was considered statistically significant.

## 3 Results

### 3.1 Efficacy and complications of radiotherapy

The total effective rate in the observation group was significantly higher than that in the control group, while the incidence of complications was lower (*P* < 0.05) (Table 2).

**Table 2:** Effect of radiotherapy and complications (case [%])

Group	Number of cases	Significance	Effective	Stability	Ineffective	Total effectiveness	Bone marrow suppression	Severe damage to adjacent organs	Total complications
Control group	44	23 (52.3)	10 (22.7)	8 (18.2)	3 (6.8)	33 (75.0)	3 (6.8)	5 (11.4)	8 (18.2)
Observation group	44	29 (65.9)	11 (25.0)	3 (6.8)	1 (2.3)	40 (90.9)	1 (2.3)	1 (2.3)	2 (4.6)
*χ* ^2^						3.938			4.062
*P*						0.047			0.044

### 3.2 Serum NLR, IL-6, and TNF-α levels

**Figure 1:**
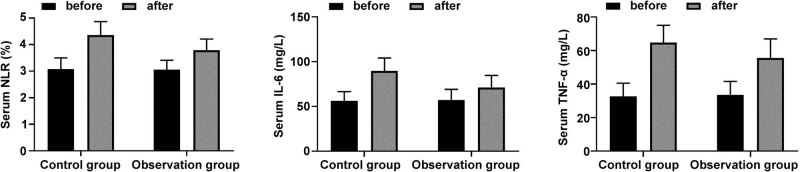
ELISA comparison of serum NLR, IL-6, and TNF-α before and after treatment.

There was no significant difference in serum NLR, IL-6, and TNF-α before radiotherapy (*P* > 0.05). The levels of NLR, IL-6, and TNF-α after radiotherapy were significantly higher than before radiotherapy, but the above indicators in the observation group were lower than those in the control group (*P* < 0.05) (Figure 1).

**Figure 2:**
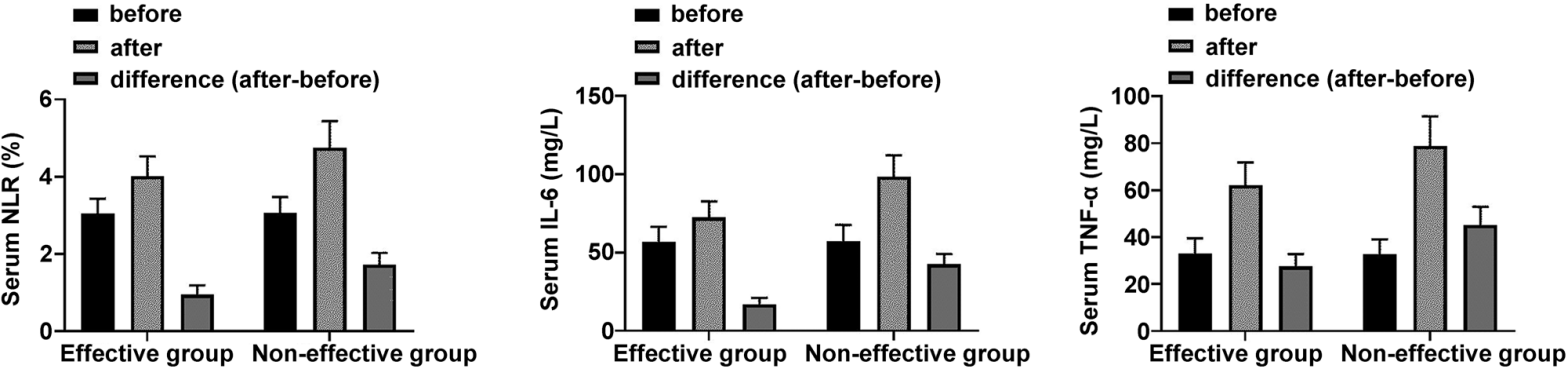
ELISA comparison of serum NLR, IL-6, and TNF-α in the effective and non-effective groups.

Patients were divided into effective group (*n* = 73) and non-effective group (*n* = 16) according to the efficacy of radiotherapy. There was no difference in serum NLR, IL-6, and TNF-α before radiotherapy (*P* > 0.05). After radiotherapy, the levels of NLR, IL-6, and TNF-α were also higher than before radiotherapy, but serum NLR, IL-6, and TNF-α levels in the effective group were significantly lower than those in the non-effective group (*P* < 0.05) (Figure 2).

### 3.3 ROC analysis

**Figure 3:**
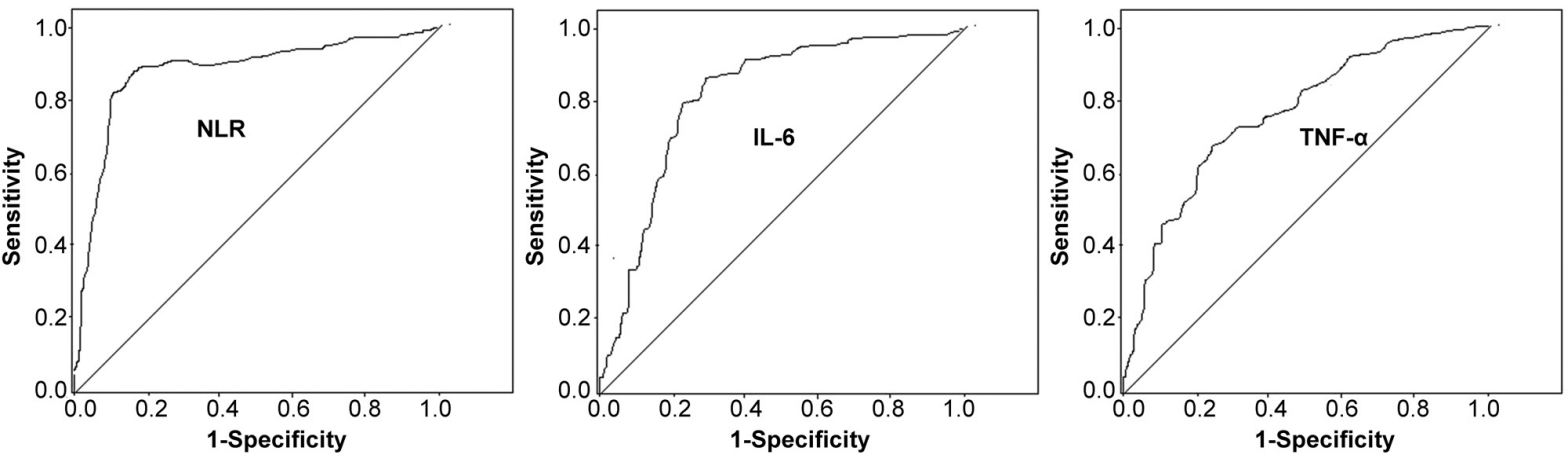
ROC analysis of serum NLR, IL-6, and TNF-α levels after radiotherapy to predict radiotherapy effect.

The specificity of serum NLR, IL-6, and TNF-α levels in predicting radiotherapy effect after ROC analysis was 0.823, 0.759, and 0.724, respectively (Table 3 and Figure 3).

**Table 3:** Serum NLR, IL-6, and TNF-α levels after radiotherapy predict the effects of radiotherapy

Indicators	AUC	95 CI	*P* values	(a) Sensitivity (%)	Specificity (%)	Cut-off values
NLR (%)	0.823	0.778–0.896	0.006	75.6	70.5	4.22
IL-6 (mg/L)	0.759	0.706–0.842	0.012	71.2	66.9	83.6
TNF-2 ∼0.842	0.724	0.659–0.813	0.017	70.8	62.3	67.5

### 3.4 Logistic regression analysis

Multiple factor logistic regression analysis was used to screen the factors affecting radiotherapy effect. T stage (with IIb as reference), target area delineation (with control group as reference), and serum NLR, IL-6, and TNF-α were all independent risk factors (*P* < 0.05) (Table 4).

**Table 4:** Logistic regression analysis screening factors affecting radiotherapy effect

Factors	*β*	Wald values	*P* values	OR values	95 CI
T stage	0.065	5.654	0.002	2.659	2.054–3.326
III	0.121	5.032	0.008	2.235	1.754–2.869
IV	0.036	5.987	0.001	2.865	2.214–3.568
Target mapping	0.254	4.865	0.012	2.006	1.235–2.568
Serum NLR	0.306	4.659	0.016	1.659	1.124–2.254
IL-6	0.352	4.427	0.022	1.327	1.065–1.857
TNF-α	0.487	4.128	0.027	1.128	1.002–1.528

## 4 Discussion

According to the results of this study, the total effective rate of radiotherapy in the observation group was significantly higher than that in the control group, while the incidence of complications was lower. It is suggested that the nasopharyngeal target localization-based approach in tumor radiotherapy holds promise for enhancing treatment efficacy and mitigating radiotherapy-related complications in patients. The delineation of the radiotherapy target area for NPC has been a subject of controversy due to the small size of the nasopharyngeal cavity, the complexity of the surrounding adjacent tissue structure, and the tumor’s biological characteristics, which make it prone to easy diffusion [13]. NPC is classified into high-risk, middle-risk, and low-risk areas. It is found that when the high-risk areas are invaded, the invasion risk of adjacent areas increases to 55.2%, while when high-risk areas are not invaded, the invasion risk of most adjacent areas is less than 10%, i.e., NPC has the characteristics of near-to-far invasion, which is the basis of international consensus on NPC target areas [14]. CTVp1 = GTVp + 5 mm (if the slope is not involved, no slope coverage is required) is based on histopathological measurements that the mean target volume of the tumor on the horizontal and long axes is 3–4 mm larger than the measured data [15]. In most Asian countries with a high incidence of NPC, inclusion of the entire nasopharyngeal cavity in CTVp1 is more common [16] CTVp2 = CTVp1 + 5 mm + the entire nasopharynx, i.e., GTVp expansion of at least 10 mm is based on tumor cells infiltrating the submucosal depth of 7.4–13.8 and expansion of 15, inevitably increasing radiation damage to adjacent tissues [17].

The study showed that NLR, IL-6, and TNF-α after radiotherapy in the two groups were significantly higher than those before radiotherapy, but the above indicators in the observation group were lower than those in the control group. It was suggested that radiotherapy itself increases the body’s inflammatory response and reduces immune function, but this damage appears to be alleviated in the observation group. Lin et al. [18] revealed that preoperative platelet count and NLR can better predict the survival rate of NPC patients receiving IMRT. According to 55.19-month follow-up records of 232 patients, preoperative ROC showed that patients with elevated NLR (>3) and platelet count (>300 × 10^9^/L) had an NLR score of 2, patients with or without an increase in NLR and AL had an NLR score of 1 or 0. Univariate analysis showed that NLR > 2.23 were not correlated with platelet count >290.5 × 10^9^/L. Multivariate analysis revealed that patients with NLR score of 0 had better 3-year disease-specific survival (*P* = 0.02), OS (*P* = 0.024), local recurrence-free survival (*P* = 0.004), and DMFS (*P* = 0.046). Further analysis also showed that NLR was an adverse prognostic indicator of 3-year failure-free survival in locally advanced NPC (*P* = 0.001). Therefore, it was suggested that NLR score rather than NLR alone or platelet count alone predicted survival in NPC patients receiving IMRT, especially those with III/IVA or in stage B. Ke et al. [19] built a prognostic model to predict the risk of distant metastasis and death in NPC patients according to serum IL-6 level and clinical stage before treatment. Increased IL-6 levels were positively correlated with 9-year OS, DFS, DMFS, and lung metastasis free survival (MFS). Model based on TNM stage and IL-6 level can predict OS, DFS, DMFS, and lung MFS of NPC patients. Jin et al. [20] explored the plasma expression of cytokines and chemokines in NPC patients after IMRT. The plasma expression of 19 cytokines and chemokines in NPC patients was higher than that in healthy individuals. Only IL-1b, IL-6, MCP-1, TNF-α, FKN, IL-12P70, IL-2, IL-5, and IP-10 suggested significant differences. However, the expression level of 19 cytokines and chemokines decreased significantly after treatment, especially IFN-γ, IL-10, IL-1b, IL-6, IL-8, MCP-1, TNF-α, VEGF, IL-17A, IL-2, IL-5, and MIP-1b. Plasma levels of IFN-γ, IL-1b, IL-6, MCP-1, TNF-α, IL-2, and IL-5 in patients with NPC were significantly increased and significantly decreased after treatment, suggesting that these cytokines and chemokines may play an important role in the occurrence and development of NPC.

The study also divided the effective and non-effective groups according to the radiotherapy effect. Serum NLR, IL-6, and TNF-α levels in the effective group were significantly lower than those in the non-effective group after radiotherapy, indicating that changes in serum NLR, IL-6, and TNF-α levels have certain significance in response to radiotherapy [21]. ROC analysis showed that the specificity of NLR, IL-6, and TNF-α in predicting radiotherapy effect was 0.823, 0.759, and 0.724, respectively. It is speculated that serum NLR, IL-6, and TNF-α levels have great potential to predict the effect of radiotherapy [22].

Finally, it was found that T stage, target area delineation, and serum NLR, IL-6, and TNF-α levels were independent risk factors. It has been confirmed that serum levels of NLR, IL-6, and TNF-α play an important role in the course of NPC radiotherapy, which may have a greater application value in predicting the radiotherapy effect [23].

To sum up, the efficacy and potential complications of radiotherapy for NPC can be influenced by the choice of target localization techniques. Changes in serum NLR, IL-6, and TNF-α levels have certain significance for predicting the effect of radiotherapy.
